# Supplementary material for: Time-series transcriptome analysis identified differentially expressed genes in broiler chicken infected with mixed Eimeria species
Source: Front Genet. 2022 Aug 8;13:886781. doi: 10.3389/fgene.2022.886781 (PMC9393255; doi:10.3389/fgene.2022.886781)
Supplement: Supplementary file 2 [file DataSheet1.ZIP › 7dpi_KEGG.Gsea.1625115961950/KEGG_FC_EPSILON_RI_SIGNALING_PATHWAY.html]

Details for gene set KEGG\_FC\_EPSILON\_RI\_SIGNALING\_PATHWAY[GSEA]

|  || Dataset | TMM\_7dpi\_gct\_format\_7dpi\_gct\_format.Class\_4dpi.cls #PC\_versus\_NC.Class\_4dpi.cls #PC\_versus\_NC\_repos |
| Phenotype | Class\_4dpi.cls#PC\_versus\_NC\_repos |
| Upregulated in class | 0 |
| GeneSet | KEGG\_FC\_EPSILON\_RI\_SIGNALING\_PATHWAY |
| Enrichment Score (ES) | -0.45702595 |
| Normalized Enrichment Score (NES) | -1.5283711 |
| Nominal p-value | 0.0106501 |
| FDR q-value | 0.11957628 |
| FWER p-Value | 0.9198 |
Table: GSEA Results Summary

  

Fig 1: Enrichment plot: KEGG\_FC\_EPSILON\_RI\_SIGNALING\_PATHWAY      
 Profile of the Running ES Score & Positions of GeneSet Members on the Rank Ordered List

  

| SYMBOL | TITLE | RANK IN GENE LIST | RANK METRIC SCORE | RUNNING ES | CORE ENRICHMENT || 1 | PLA2G2E | na | 298 | 0.942 | 0.0022 | No |
| 2 | PLA2G5 | na | 299 | 0.942 | 0.0292 | No |
| 3 | MAPK11 | na | 678 | 0.646 | 0.0162 | No |
| 4 | HRAS | na | 1342 | 0.420 | -0.0272 | No |
| 5 | VAV3 | na | 1390 | 0.408 | -0.0194 | No |
| 6 | RAC1 | na | 2998 | 0.126 | -0.1502 | No |
| 7 | MAP2K2 | na | 3435 | 0.070 | -0.1846 | No |
| 8 | MAP2K3 | na | 3856 | 0.021 | -0.2192 | No |
| 9 | MAP2K4 | na | 4132 | -0.010 | -0.2419 | No |
| 10 | PLA2G6 | na | 4448 | -0.045 | -0.2669 | No |
| 11 | BTK | na | 4962 | -0.100 | -0.3070 | No |
| 12 | RAF1 | na | 5142 | -0.116 | -0.3186 | No |
| 13 | MAP2K1 | na | 5188 | -0.120 | -0.3189 | No |
| 14 | PRKCD | na | 5230 | -0.124 | -0.3188 | No |
| 15 | MAPK13 | na | 5231 | -0.124 | -0.3152 | No |
| 16 | RAC3 | na | 6076 | -0.210 | -0.3798 | No |
| 17 | PLCG1 | na | 6241 | -0.228 | -0.3869 | No |
| 18 | MAPK8 | na | 6464 | -0.250 | -0.3983 | No |
| 19 | GRB2 | na | 6686 | -0.274 | -0.4089 | No |
| 20 | AKT3 | na | 7033 | -0.312 | -0.4289 | No |
| 21 | PLA2G4A | na | 7107 | -0.320 | -0.4258 | No |
| 22 | KRAS | na | 7206 | -0.330 | -0.4245 | No |
| 23 | NRAS | na | 7474 | -0.358 | -0.4365 | No |
| 24 | PLA2G4B | na | 7552 | -0.367 | -0.4324 | No |
| 25 | MAPK12 | na | 7612 | -0.373 | -0.4266 | No |
| 26 | MAPK9 | na | 7648 | -0.377 | -0.4187 | No |
| 27 | RAC2 | na | 7904 | -0.409 | -0.4283 | No |
| 28 | PDPK1 | na | 8107 | -0.435 | -0.4327 | No |
| 29 | AKT1 | na | 8399 | -0.472 | -0.4435 | Yes |
| 30 | PIK3CG | na | 8478 | -0.481 | -0.4362 | Yes |
| 31 | MAPK14 | na | 8502 | -0.484 | -0.4242 | Yes |
| 32 | PRKCA | na | 8704 | -0.513 | -0.4262 | Yes |
| 33 | SOS1 | na | 8869 | -0.533 | -0.4246 | Yes |
| 34 | PRKCE | na | 8985 | -0.547 | -0.4185 | Yes |
| 35 | FCER1G | na | 9176 | -0.578 | -0.4178 | Yes |
| 36 | PIK3R2 | na | 9597 | -0.645 | -0.4344 | Yes |
| 37 | SYK | na | 9655 | -0.655 | -0.4204 | Yes |
| 38 | PLA2G12B | na | 9771 | -0.669 | -0.4107 | Yes |
| 39 | PRKCB | na | 9867 | -0.685 | -0.3990 | Yes |
| 40 | MAPK3 | na | 10320 | -0.774 | -0.4146 | Yes |
| 41 | PIK3CB | na | 10335 | -0.777 | -0.3934 | Yes |
| 42 | LCP2 | na | 10364 | -0.782 | -0.3733 | Yes |
| 43 | PLCG2 | na | 10697 | -0.853 | -0.3765 | Yes |
| 44 | PIK3CD | na | 10707 | -0.855 | -0.3527 | Yes |
| 45 | PIK3CA | na | 10920 | -0.912 | -0.3442 | Yes |
| 46 | PIK3R5 | na | 10941 | -0.918 | -0.3195 | Yes |
| 47 | PIK3R3 | na | 11045 | -0.949 | -0.3008 | Yes |
| 48 | MAP2K6 | na | 11185 | -0.996 | -0.2838 | Yes |
| 49 | PIK3R1 | na | 11265 | -1.023 | -0.2610 | Yes |
| 50 | LYN | na | 11314 | -1.043 | -0.2350 | Yes |
| 51 | SOS2 | na | 11362 | -1.063 | -0.2084 | Yes |
| 52 | FYN | na | 11389 | -1.076 | -0.1796 | Yes |
| 53 | INPP5D | na | 11445 | -1.096 | -0.1527 | Yes |
| 54 | VAV2 | na | 11979 | -1.860 | -0.1438 | Yes |
| 55 | GAB2 | na | 12006 | -2.362 | -0.0781 | Yes |
| 56 | MAPK10 | na | 12008 | -2.724 | 0.0002 | Yes |
Table: GSEA details [plain text format]

  

Fig 2: KEGG\_FC\_EPSILON\_RI\_SIGNALING\_PATHWAY      
 Blue-Pink O' Gram in the Space of the Analyzed GeneSet

  

Fig 3: KEGG\_FC\_EPSILON\_RI\_SIGNALING\_PATHWAY: Random ES distribution      
 Gene set null distribution of ES for **KEGG\_FC\_EPSILON\_RI\_SIGNALING\_PATHWAY**
